# Attitudes of Australian dermatologists on the use of genetic testing: A cross-sectional survey with a focus on melanoma

**DOI:** 10.3389/fgene.2022.919134

**Published:** 2022-10-24

**Authors:** Clare A. Primiero, Amy M. Baker, Courtney K. Wallingford, Ellie J. Maas, Tatiane Yanes, Lindsay Fowles, Monika Janda, Mary-Anne Young, Amy Nisselle, Bronwyn Terrill, Jason M. Lodge, Jane M. Tiller, Paul Lacaze, Hayley Andersen, Gemma McErlean, Erin Turbitt, H. Peter Soyer, Aideen M. McInerney-Leo

**Affiliations:** ^1^ The University of Queensland Diamantina Institute, Dermatology Research Centre, The University of Queensland, Brisbane, QLD, Australia; ^2^ Discipline of Genetic Counselling, Graduate School of Health, University of Technology Sydney, Sydney, NSW, Australia; ^3^ Genetic Health Queensland, Royal Brisbane and Women’s Hospital, Brisbane, QLD, Australia; ^4^ Centre for Health Services Research, The University of Queensland, Brisbane, QLD, Australia; ^5^ Kinghorn Centre for Clinical Genomics, Garvan Institute of Medical Research, Sydney, NSW, Australia; ^6^ Medicine and Health, University of New South Wales, Sydney, NSW, Australia; ^7^ Australian Genomics Health Alliance, Melbourne, VIC, Australia; ^8^ Murdoch Children’s Research Institute, Department of Paediatrics, The University of Melbourne, Parkville, VIC, Australia; ^9^ School of Education, The University of Queensland, Brisbane, QLD, Australia; ^10^ Public Health Genomics, Department of Epidemiology and Preventive Medicine, School of Public Health and Preventive Medicine, Monash University, Melbourne, VIC, Australia; ^11^ Melanoma and Skin Cancer Advocacy Network, Carlton, VIC, Australia; ^12^ SWS Nursing and Midwifery Research Alliance, Ingham Institute for Applied Medical Research, Liverpool, NSW, Australia; ^13^ School of Nursing, University of Wollongong, Wollongong, NSW, Australia; ^14^ Department of Dermatology, Princess Alexandra Hospital, Brisbane, QLD, Australia

**Keywords:** genetics, genomics, dermatology, mainstreaming, familial melanoma

## Abstract

**Background:** Melanoma genetic testing reportedly increases preventative behaviour without causing psychological harm. Genetic testing for familial melanoma risk is now available, yet little is known about dermatologists’ perceptions regarding the utility of testing and genetic testing ordering behaviours.

**Objectives:** To survey Australasian Dermatologists on the perceived utility of genetic testing, current use in practice, as well as their confidence and preferences for the delivery of genomics education.

**Methods:** A 37-item survey, based on previously validated instruments, was sent to accredited members of the Australasian College of Dermatologists in March 2021. Quantitative items were analysed statistically, with one open-ended question analysed qualitatively. *Results:* The response rate was 56% (256/461), with 60% (153/253) of respondents between 11 and 30 years post-graduation. While 44% (112/252) of respondents agreed, or strongly agreed, that genetic testing was relevant to their practice today, relevance to future practice was reported significantly higher at 84% (212/251) (*t =* -9.82, *p* < 0.001). Ninety three percent (235/254) of respondents reported rarely or never ordering genetic testing. Dermatologists who viewed genetic testing as relevant to current practice were more likely to have discussed (*p* < 0.001) and/or offered testing (*p* < 0.001). Respondents indicated high confidence in discussing family history of melanoma, but lower confidence in ordering genetic tests and interpreting results. Eighty four percent (207/247) believed that genetic testing could negatively impact life insurance, while only 26% (63/244) were aware of the moratorium on using genetic test results in underwriting in Australia. A minority (22%, 55/254) reported prior continuing education in genetics. Face-to-face courses were the preferred learning modality for upskilling.

**Conclusion:** Australian Dermatologists widely recognise the relevance of genetic testing to future practice, yet few currently order genetic tests. Future educational interventions could focus on how to order appropriate genetic tests and interpret results, as well as potential implications on insurance.

## 1 Introduction

Availability of genetic testing for hereditary cancers has increased in the last 2 decades (Collins and McKusick, 2001; Brittain et al., 2017; Williams, 2019). However, the integration of genetic testing into routine clinical practice remains low (Cornel, 2019; Johnson et al., 2020; Best et al., 2021). Traditionally, clinical genetic testing is offered by a qualified genetic counsellor or clinical geneticist (Badenas et al., 2012). Recently, given the rising demand for genomic services (Frost et al., 2019), and the limited clinical genetic workforce (Hoskovec et al., 2018; McCuaig et al., 2018), genetic care has been integrated into non-genetics clinics in a process known as “mainstreaming”. This has increasingly occurred in oncology care for hereditary breast, ovarian, colorectal and endometrial cancer (McCuaig et al., 2018; O'Shea et al., 2021). Previous mainstreaming interventions have demonstrated significant positive outcomes such as increased access to genetic testing and identification of hereditary cancer cases (Heald et al., 2013; Miesfeldt et al., 2018), as well as decreased health care costs (George et al., 2016; Plaskocinska et al., 2016; Kemp et al., 2019) and wait times for appointments and results (Heald et al., 2013; George et al., 2016; Plaskocinska et al., 2016; Kentwell et al., 2017; Rahman et al., 2019; Richardson et al., 2020; Rumford et al., 2020). This suggests that non-genetics practitioners are capable of understanding and ordering genetic tests for their patients.

We propose that there is an opportunity for mainstreaming familial melanoma genetic testing into dermatology practice as the genetics are well understood (Leachman et al., 2017; Abdo et al., 2020; Toussi et al., 2020). *CDKN2A*, accounts for 20%–40% of hereditary melanoma cases and 90% of positive results (Aoude et al., 2015; Potrony et al., 2015; Read et al., 2016). However, several other genes are associated with familial melanoma including *BAP1, BRCA2, CDK4, MC1R, MITF, POLE, POT1, PTEN, RB1, TERT* and *TP53* (Ribeiro Moura Brasil Arnaut et al., 2021; Maas et al., 2022). A pathogenic mutation in these genes can incur a lifetime risk of melanoma of up to 84% (Box et al., 2001), compared to the national Australian average of approximately 5% (Duffy et al., 2019). Recent reviews on the impact of familial melanoma genetic testing have shown a positive effect on protective behaviours (Primiero et al., 2021a), without adverse psychological outcomes (Primiero et al., 2021b). Longitudinal studies have reported improved Sun protection including decreased sunburns and Sun exposure, and increased adherence to regular clinical skin examinations, for up to 2 years after receiving genetic testing for *CDKN2A* mutations (Aspinwall et al., 2008; Aspinwall et al., 2014; Stump et al., 2020). Not only is increased screening/surveillance in high-risk individuals cost affective (Guitera et al., 2021) but it is associated with earlier detection and improved outcomes i.e., morbidity and mortality (Gordon and Rowell, 2015). Dermatologists are well placed to identify patients who may benefit from genetic testing (Zhou et al., 2021) and customise subsequent screening recommendations accordingly (Zhou et al., 2021). Given the success of previous mainstreaming efforts in other specialist settings (Talwar et al., 2017; Kohut et al., 2019), we envision that genetic testing for familial melanoma could feasibly become standard dermatological practice. We note that a pilot program to upskill clinicians to provide genetic testing for familial melanoma is currently being trailed in a small cohort of clinicians (Primiero et al., 2022). Future integration of a mainstreaming intervention should be guided by an implementation theory framework, such as Procter’s implementation outcome framework (Proctor et al., 2011), or the Consolidated Framework for Implementation Research (CFIR) (Damschroder et al., 2009). Both frameworks describe the importance of engaging key stakeholders to gauge receptibility, attitudes and experience of a new intervention. They can also be used to evaluate the efficacy and sustainability of the intervention.

Previous studies investigating attitudes and preparedness for genetic testing in other settings have largely targeted general (family) practitioners (Carroll et al., 2009; Nippert et al., 2011a; Bouhnik et al., 2017; Diamonstein et al., 2018; Carroll et al., 2019; Haga et al., 2019; Harding et al., 2019; DeLuca et al., 2020), obstetricians-gynaecologists (Nippert et al., 2011a; Douma et al., 2016; Bouhnik et al., 2017; Diamonstein et al., 2018; Kathrens-Gallardo et al., 2021), oncologists (Douma et al., 2016; Bouhnik et al., 2017; Chow-White et al., 2017) and paediatricians (Nippert et al., 2011a; Johnson et al., 2017; Diamonstein et al., 2018). To date, evaluation of dermatologists’ views on genomic medicine has been limited to paediatric dermatologists (Shagalov et al., 2017), trainees (Murphy, 2015), and fellowship program directors (Torre et al., 2018). These studies report an increasing recognition of the relevance of genetic testing to their speciality, but also a deficit in training and education (Chow-White et al., 2017; Johnson et al., 2017; Diamonstein et al., 2018; Torre et al., 2018; Crellin et al., 2019; Harding et al., 2019). The current study evaluates the present perception, use, and confidence in using genetic testing in dermatology practice in Australia, including preferences for the delivery of future education interventions. In this survey we refer to both “genetic/genomic” testing. However, in this manuscript we will refer to both as “genetic testing”.

## 2 Methods

A cross-sectional survey was posted to all Australian Dermatologists to capture current confidence and attitudes regarding genetic testing, and guide future education interventions. Specifically, we were interested in predictors of genetic confidence, and the relationship between attitudes towards genetic testing and current practice.

The capability, opportunity, and motivation model of behaviour (COM-B) was the guiding framework in the survey design. The COM-B model considers three interacting constructs relating to the uptake of a new practice including; 1. Capability (knowledge and competence), 2. Opportunity (resources), and 3. Motivation (perceived benefit of genetic testing) (Michie et al., 2011; McClaren et al., 2020). This study followed the Strengthening the Reporting of Observational Studies in Epidemiology (STROBE) reporting guideline.

### 2.1 Survey development

The survey was structured into four sections, using previously validated scales and purpose-designed items. Section 1 measured attitudes regarding the relevance of genetic testing to current and future dermatology practice, using a five-point Likert scale. These questions were adapted from an original 4-item instrument developed for community-based physicians, by using only the first two items, and replacing “genomic medicine” with “genetic testing” (Reed et al., 2016). Section 2 captured genetic experience and confidence domains with three validated scales: an 8-item scale, adapted from a 14-item validated scale (Bouhnik et al., 2017) by referring to “genetic testing for melanoma”, instead of *BRCA1* or *BRCA2* testing, and selecting questions relevant to the potential benefits and limitations of genetic testing for familial melanoma (Agree/Disagree); a 5-item scale (Culver et al., 2001) assessing whether respondents had previously performed certain genetic skills in practice (Yes/No); and a 11-item, five-point Likert Scale (Not at all confident/Very confident) (Reed et al., 2016) with one additional question regarding patient consent, required respondents to rate their confidence in performing specific genetic skills (Cronbach’s Alpha = 0.90). Section 3 used purpose-designed questions to capture education preferences (rank 1–5), previous continuing education in genetics/genomics (Yes/No), and perceived usefulness of previous training (Scale 1–10, where 1 was “Not at all useful” and 10 was “Very useful”). Section 4 collected demographics, frequency of ordering genetic testing, and patient enquiries about genetic testing. Finally, an open-ended question invited qualitive feedback regarding their attitudes towards genetic testing in dermatology (see Supplementary Material S1 for survey). The final survey was piloted in two dermatologists for feedback prior to wider distribution.

### 2.2 Study population

In March 2021 a survey was posted to all Australian members of the Australasian College of Dermatologists (ACD), excluding trainees as no site of employment was available for them. Contact details were accessible for only one member in New Zealand, therefore only Australian members were included. A paper-based survey was administered, to allow the inclusion of an AUS$10 cash incentive, as incentives have previously been shown to increase physician response rates (James et al., 2011; Noel and Huang, 2019; Demeshko et al., 2020). The ACD included a notification in their e-newsletter prior to the survey mailing. Two weeks after survey mailing, the ACD emailed the membership to remind them to complete the survey and provided a link to an online version of the survey. The online survey tool was hosted by The University of Queensland using Checkbox^®^. A waiver of consent was obtained as consent was considered implied if participants chose to complete the survey. Human Research Ethics was approved by The University of Queensland HREC (ref: 2020002658) and ratified by the University of Technology Sydney HREC (ref: ETH20-5671).

### 2.3 Data analysis

Surveys were transcribed in a text-delimited format and analysed using IBM SPSS Statistical Package (Corp, 2020). Descriptive statistics were used to summarise participant characteristics. Confidence ratings for individual genetic skills were consolidated to create a cumulative average genetic confidence score (out of 5). Simple linear regression identified variables associated with overall genetic confidence. An unpaired Student t-test compared attitudes towards relevancy of genetic testing in current and future practice. Chi-squared analysis (or Fisher’s Exact Test if the “n” was <5 in any of the cells) compared current and future attitudes towards the relevancy of genetic testing and the likelihood of having previously discussed, offered, ordered, or referred for genetic tests. *p*-values of <0.05 were considered significant and reported with 95% confidence intervals (CI). Qualitative responses to the open-ended question were evaluated using thematic content analysis methods (Bengtsson, 2016) employing NVivo 12 Plus to manage transcripts, as well as code and compile inferences from responses.

## 3 Results

### 3.1 Sample demographics

A total of 494 surveys were posted; 34 were returned unopened and deemed ineligible. Of the 461 eligible surveys, 247 were returned, and a further nine were completed online, giving a response rate of 56%. Demographic characteristics of respondents are displayed in Table 1. Most respondents were from either New South Wales (35%), Victoria (22%), or Queensland (20%), which encompass Australia’s three most populous cities. Over half the respondents were either 11–20 years (33%), or 21–30 years post-graduation (27%). A third of respondents reported having a sub-specialty.

**TABLE 1:** Participant demographic characteristics (*n* = 256).

Variable	n (%)
**Survey format**	**256**
Paper	247 (96)
Online	9 (4)
**Location (within Australia/New Zealand)**	**255**
New South Wales	87 (34)
Victoria	59 (23)
Queensland	54 (21)
Western Australia	28 (11)
South Australia	21 (8)
Australian Capital Territory	3 (<1)
Tasmania	2 (<1)
Northern Territory	1 (<1)
**Years since graduated from medical school**	**253**
1–10 years	15 (6)
11–20 years	86 (34)
21–30 years	67 (26)
31–40 years	47 (19)
40 > years	38 (15)
**Reported Sub-Speciality in Dermatology**	**250**
Yes	**84 (34)**
Paediatric Dermatology	26 (31)
Mohs and Dermatologic Surgery	21 (25)
Oncology Dermatology	8 (10)
Other	29 (35)

Bold values represent the n that provided a response to that subset of questions.

### 3.2 Current role of genetic testing in dermatology

When asked if they have ever discussed, offered, or referred people for genetic testing, the majority (96%, n = 246) reported having previously discussed it; 91% had offered it, 89% had referred patients for it, and 60% had previously ordered tests. When asked how frequently genetic testing was ordered, 29% reported never ordering, 63% ordered rarely (≤5 times a year), 6% often (≥ once a month), and only 1% (n = 3) routinely (≥ once a week). Only 19% indicated they had the “necessary services and staff” to offer genetic testing. Respondents who indicated they “never” or “rarely” ordered genetic testing, were subsequently asked, “why not?”, and provided with a list of common reasons to select from, including an open field for “other”. Of note, 22% of respondents reported that their lack of confidence contributed to their infrequent ordering. Results are displayed in Table 2.

**TABLE 2:** Current role of genomic medicine for any dermatological condition, including previously-performed genetic tasks, frequency of ordering genetic/genomic testing, and reasons for infrequent ordering.

Previous performed genetic tasks	n = 256 (%)
I have discussed genetic testing with my patients	246 (96)
I have offered genetic testing to my patients	232 (91)
I have ordered genetic testing for my patients	153 (60)
I have referred my patients to clinical genetic services	226 (89)
I have the necessary resources to offer genetic testing to patients	49 (19)
Frequency of ordering genetic/genomic testing	**n = 254 (%)**
Never	74 (29)
Rarely (≤5 times a year)	161 (63)
Often (≥ once a month	16 (6)
Routinely (≥ once a week)	3 (1)
Reasons for not ordering genetic testing more frequently	**n = 235 (%)**
Not relevant to my practice	81 (34)
I do not feel confident	51 (22)
It is not my role	20 (9)
I do not have access to a genetic service	28 (12)
I do not have time	2 (<1)
Other (categorised below)	65 (28)
*Refer patients to genetic services*	29 (12)
*Genetic testing irrelevant for my patients*	23 (10)
*Lack of information/knowledge on available genetic testing*	6 (3)
*Concerns regarding costs*	5 (2)
*Lack of time to discuss/order genetic testing*	2 (<1)
*Concerns about impact on insurance*	1 (<1)

Bold values represent the n that provided a response to that subset of questions.

### 3.3 Attitudes towards genetic testing in dermatology

While 44% of respondents agreed/strongly agreed that genetic testing was relevant to current practice, 85% agreed/strongly agreed that genetic testing will be relevant to future practice (*t* = -9.82, *p* < 0.001).

Participants were asked whether they agreed or disagreed with possible benefits and limitations of genetic testing for melanoma. All statements relating to benefits of genetic testing had very strong agreeance, with at least 90% agreeing that genetic testing for melanoma could be of value to the patient, their family, for management decisions, and/or to improve primary/secondary prevention (Table 3).

**TABLE 3:** Respondent’s perceptions on benefits, and limitations of genetic testing in dermatology.

Perceptions on genetic testing for melanoma in dermatology	“Yes”/total (%)^a^
*Benefits of Genetic testing*	
Genetic testing for melanoma could be of value to the patient	235/252 (93%)
Genetic testing for melanoma could be of value to the patient’s family	242/253 (96%)
Genetic testing for melanoma could be of value in informing management decisions	226/252 (90%)
Genetic testing for melanoma could be of value in improving primary/secondary prevention	224/250 (90%)
*Limitations of Genetic testing*	“Agree”/total (%)^b^
Genetic testing for melanoma could limit the patient’s private health insurance coverage	196/247 (79%)
Genetic testing for melanoma could limit the patient’s life insurance coverage	207/247 (84%)
Awareness that a moratorium was introduced on the use of genetic testing in life insurance underwriting in July 2019	63/244 (26%)
Genetic testing for melanoma could stigmatise the patients as a “worried well” person	145/252 (58%)

^a^ Yes/No options. Number of those answering “Yes” divided by total number who answered the question.

^b^ Agree/Disagree options. Number of those answering “Agree” divided by total number who answered the question.

Most respondents also agreed with the statements on perceived limitations of genetic testing; 79% believed it could limit access to private health insurance, 84% agreed that it could limit access to life insurance, and 58% felt that melanoma genetic testing could stigmatise individuals as a “worried well”.

Participants who viewed genetic testing as relevant to their current practice were significantly more likely to have discussed (*p* < 0.001, Fisher’s Exact Test) or offered it to patients, X^2^ (1, *N* = 256) = 17.5, *p* < 0.001, ordered testing, X^2^ (1, *N* = 256) = 6.8, *p* = 0.009, and referred patients to genetic services, X^2^ (1, *N* = 256) = 9.3, *p* = 0.002.

### 3.4 Confidence in basic genetic tasks

**FIGURE 1:**
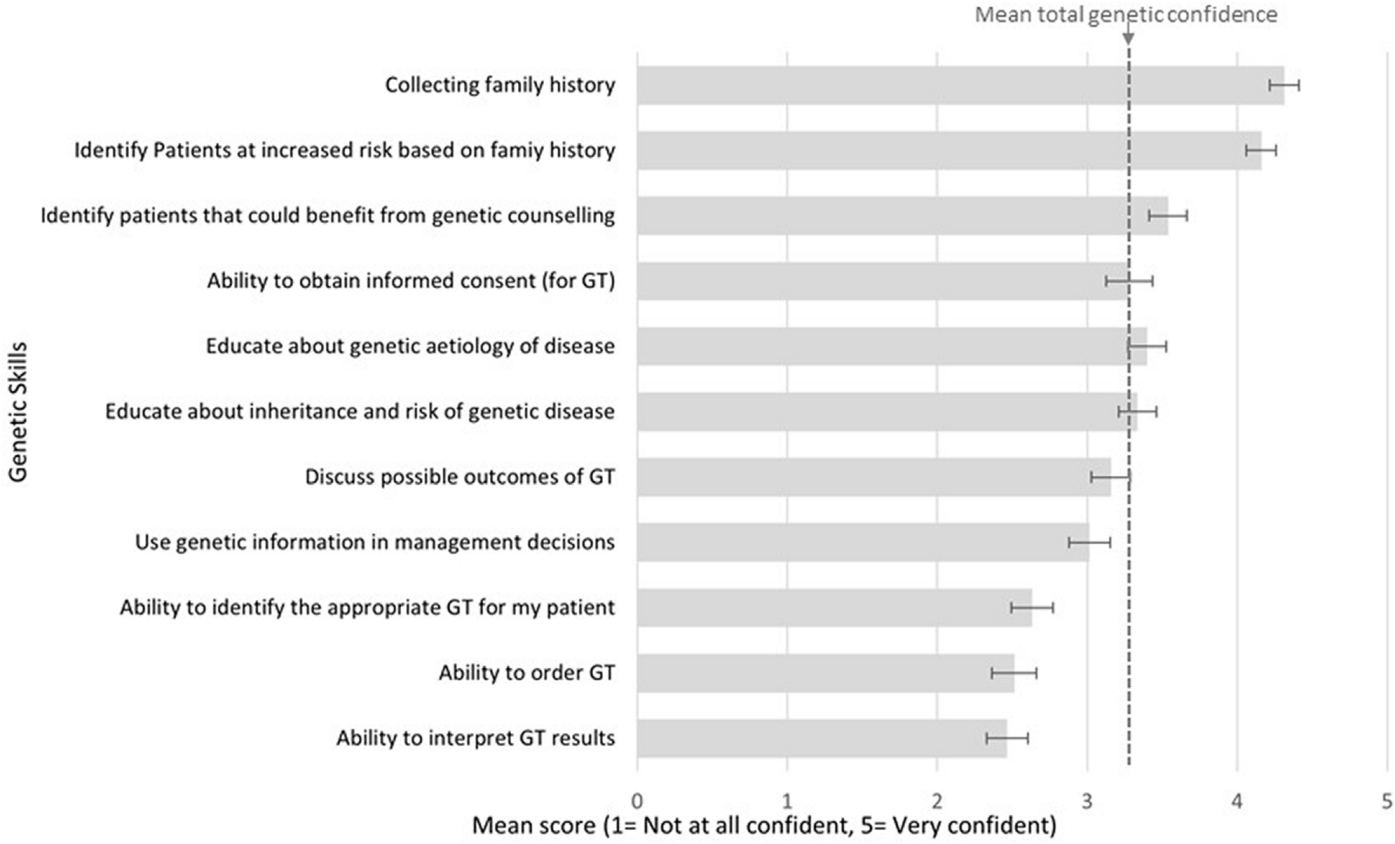
Respondents reported confidence in basic genetic skills.

High confidence was reported for collecting family history (mean score 4.3/5; CI 4.2–4.4), and identifying patients at risk of a hereditary condition (4.2/5; CI 4.1–4.3). Moderate confidence was reported for identifying patients who could benefit from genetic testing (3.5/5; CI 3.4–3.7), educating patients on genetic aetiology of disease (3.4/5; CI 3.3–3.5), obtaining informed consent (3.3/5; CI 3.1–3.4), discussing possible outcomes of genetic testing (3.2/5; CI 3.0–3.3), and using genetic information in management decisions (3.0/5; CI 2.9–3.2). Lower confidence was reported for identifying appropriate tests (2.6/5; CI 2.5–2.8), test ordering (2.5/5; CI 2.4–2.7), and interpreting results (2.5/5; CI 2.3–2.6). A cumulative average was calculated to provide an overall genetic skills confidence score (3.3/5; CI 3.2–3.3). Confidence results are displayed in Figure 1.

### 3.5 Previous education and preferences for future training

Prior experience with continuing education in genetics, and its relative usefulness are displayed in Table 4. Less than a quarter of respondents reported previous education in genetics, but the usefulness of these educational interventions was rated highly (6.9–7.3 out of 10).

**TABLE 4:** Prior experience with genetic/genomic education and training, and preferences for future learning.

Education in genetics/genomics^a^	n = 253 (%)	Usefulness mean^b^ (CI)
Completed a unit of study as part of an award course (degree/certificate/diploma)	55 (22)	7.1 (6.5–7.6)
Completed a short course on genetics/genomics	30 (12)	7.3 (6.8–7.9)
Completed training regarding the ethical considerations of genetics/genomics testing or research	54 (21)	6.9 (6.2–7.6)
Preferences for future learning modalities in genetics^c^	**n = 141** **^d^** **(%)**	**Average score** **^e^**
Face-to-face courses/tutorials	42 (30)	2.4
A “hotline” to talk to genetics professional	38 (27)	2.9
Online courses/tutorials	34 (24)	2.6
Experiential (observation/immersion in cases)	17 (12)	3.5
Printed material	10 (7)	3.6

^a^ The number and percent of respondents who have completed previous education in genomics, and its perceived usefulness (scored 1 to 10, with 10 indicating “very useful”).

^b^ The mean score from 1 to 10, selected by respondents (with 10 indicating “very useful”).

^c^ The number and percent of respondent’s highest ranked preference for future learning activities used to upskill in genomic medicine.

^d^ Unclear wording of question meant that only 141 individuals accurately completed this question.

^e^ The average cumulative score, a lower score indicative of most preferred (calculated by the sum of ranked values, divided by the number of values).

Bold values represent the n that provided a response to that subset of questions.

Respondents were asked to rank their preference from 1 to 5 (with one being the most preferred option) for the delivery of future education initiatives in genomic medicine. For the 141 who completed this question correctly, the most preferred learning modality was face-to-face courses (42/141), followed by a hotline for genetics advice (38/141), online courses (34/141), experiential (17/141), and lastly, printed material (10/141). When averaging the ranked score for each modality the order of preference changed only for online courses (third to second preference) and hotline for advice (second to third preference) (Table 4).

### 3.6 Variables associated with dermatologists’ confidence

Linear regression was used to identify independent variables associated with dermatologists’ overall confidence in their genetic skills (Table 5).

**TABLE 5:** Simple linear regression assessing independent predictors of dermatologists’ overall confidence in genetic skills. The dependent variable is the overall average confidence score.

	B	95% CI	β	*p*-value	*R* ^2^	F
***Characteristics and Attitude towards genetic testing***						
Years since graduating medical school	-0.05	-0.13 – 0.03	-0.07	0.251	0.01	1.32
Having a sub-speciality	0.27	0.08–0.46	0.17	**0.007**	0.03	7.50
Relevance of genetic testing to current practice	0.12	0.04–0.20	0.19	**0.003**	0.04	9.09
Relevance of genetic testing to future practice	0.06	-0.3 – 0.16	0.09	0.18	0.01	1.85
***Genetic Skills Performed***						
Discussed genetic testing with patients	0.74	0.48–1.00	0.33	**<0.001**	0.11	31.73
Offered genetic testing to patients	0.55	0.34–0.76	0.31	**<0.001**	0.09	26.28
Ordered genetic testing for at least one patient	0.50	0.32–0.68	0.33	**<0.001**	0.12	30.27
Referred to genetic services for genetic testing	0.35	0.06–0.65	0.15	**0.02**	0.02	5.46
***Continued Genetic Education***						
Award unit of study on genomics	0.17	-0.06 – 0.39	0.09	0.149	0.01	2.09
Short course on genomics	0.43	0.15–0.72	0.18	**0.003**	0.04	9.05
Training on ethical considerations of genomic research	0.52	0.30–0.74	0.28	**<0.001**	0.08	21.34

β = standardised regression coefficient.

B = unstandardised regression coefficient.

Bold values highlight significant results.

No significant relationship was found between years since graduating from medical school, however having a sub-speciality significantly predicted genetic confidence (β = 0.17, *p* = 0.007). Perceived relevance of genetic testing to current practice significantly predicted confidence (β = 0.19, *p* = 0.003), however perceived relevance to future practice was not significant. Genetic confidence was significantly predicted by having previously discussed (β = 0.33, *p* < 0.001), offered (β = 0.31, *p* < 0.001), ordered (β = 0.33, *p* < 0.001), or referred patients for genetic testing (β = 0.15, *p* = 0.02). When continued education variables were assessed, an “award unit of study on genomics’ was not associated with overall confidence, but completing a short course (β = 0.18, *p* = 0.003) or receiving training on ethical considerations (β = 0.28, *p* < 0.001) significantly predicted genetic confidence.

### 3.7 Qualitative feedback

Almost a third of participants (n = 81) provided a response to open-ended question *“Do you have any additional thoughts regarding genetic testing in dermatology, which you would like to share?”* Half (n = 41) identified the need for further training, education, and/or resources for providing genetic testing in clinical practice. Specific suggestions included requests for the College (ACD) to provide/advertise a short course on genetics, presentation updates at the Annual Scientific Meeting, and online Continuing Professional Development modules. There were several recommendations for a “*cheat sheet*”, or “*flip book*” providing clinicians with core information on tests, testing criteria, genetic counselling discussion points, test ordering and result interpretation. A few respondents expressed a need for information on relevant costs and Medicare rebates for genetic tests. Twelve individuals indicated that they are already referring patients for genetic testing, with three respondents expressing a preference to continue to refer patients rather than provide genetic testing directly. Ten comments stated the need for greater access to genetic services. Just five individuals commented that genetic testing was not useful or needed in dermatology. Five further respondents expressed concerns about costs, genetic discrimination regarding insurance, and concerns of patient anxiety.

## 4 Discussion

To our knowledge, this is the first cross-sectional survey to comprehensively examine how dermatologists are currently using genetic testing in practice, including confidence in ordering genetic tests, attitudes towards the utility of testing, and preferences for education and training. Respondents were more likely to believe genetic testing would be relevant to future practice than it was currently. This finding is consistent with a recent study of Australian non-genetic clinicians which reported that genetic testing would become increasingly important in the future (McClaren et al., 2020; Nisselle et al., 2021). Furthermore, respondents in this study were significantly more likely to have previously discussed, offered or ordered genetic testing for patients when they viewed genetic testing as relevant to their current practice. When combined with qualitative feedback, which expressed a high demand for further training in genetic medicine, these findings offer a strong indicator of motivation, a key component of the COM-B (capability, opportunity and motivation) model for behaviour change (Michie et al., 2011).

Questions exploring perceptions regarding genetic testing identified a knowledge gap regarding the potential impact on insurance in Australia. Less than a third of respondents were aware of the 2019 Australian moratorium on the use of genetic testing results in insurance underwriting (Tiller et al., 2021a). Furthermore, 79% incorrectly considered that genetic testing could impact a patient’s ability to access private health insurance, as this is not based on a risk assessment in Australia (Tiller et al., 2020). In contrast, life insurance can incorporate genetic test results in an underwriting process (Tiller et al., 2017). The moratorium prohibits life insurers from requesting genetic test results on new policies < AUS$500,000 (Tiller et al., 2021b). While this protection falls short of international standards, where some countries explicitly prohibit the use of genetic information by life insurers (Rothstein, 2018), this is still important information for dermatologists to be aware of when offering testing.

The first construct in the COM-B model relates to capability, that is, possessing the necessary knowledge and skills to perform the task (Michie et al., 2011). This survey has been valuable in illustrating which genetic tasks dermatologists already feel confident in, so as to inform the focus of future educational interventions. It is not surprising that confidence was high for identifying individuals at increased risk of a hereditary condition and recording family history. Such processes have been relevant to medical practice generally (Scheuner et al., 1997), and dermatology specifically (Rees, 1992) for an extended period. Lowest confidence was reported for tasks related directly to genetic testing and counselling, such as ordering tests and interpreting the results. These findings are congruent with similar surveys of GPs, oncologists (Demeshko et al., 2020) and obstetricians/gynaecologists (Nippert et al., 2011b). According to the COM-B model, failure to address the low confidence in genetic tasks would negatively affect successful uptake of providing genetic testing by clinicians.

Any training initiatives for dermatologists to provide genetic testing would need to cover the challenges of informed consent and interpreting and explaining test results. If a dermatologist has a patient test positive for a genetic test, they are advised to report this result to the patient and then refer them to clinical genetic services for further discussion regarding appropriate screening for non-cutaneous cancers and to arrange testing for at-risk family members. Any individuals found to carry mutations through this testing process would then be referred back to the dermatologist for appropriate screening.

An important objective of this survey was to understand the impact of past genomic education/training to guide future interventions. It was encouraging to find that completing a short course in genomics or receiving training on ethical considerations of genomic research positively predicted overall confidence in performing genetic tasks. While completing a unit/subject as part of an award course was not associated with confidence, this is not unexpected, as such genomic units are usually undertaken during early undergraduate degrees, and therefore were more than 2 decades ago for most respondents. Preferred learning modalities for future genetic education included face-to-face training, a hotline to a genetic professional, and an online course. Face-to-face courses provide opportunity for group training in an interactive setting and have previously demonstrated positive impacts on attitudes and behaviours (Cornel, 2019). A genetic professional hotline is a “just in time” method which has recently been implemented for non-genetic clinicians as an informal education strategy (Carroll et al., 2019). Online courses provide a fast and accessible educational tools, that cater to physicians unable to commit to extensive programs and democratise access to clinicians in rural/remote settings (Casebeer et al., 2010; Freeley, 2020).

The three intertwining factors of the COM-B model (*Capability, Opportunity,* and *Motivation*) are imperative to the successful implementation of new interventions. The survey results have clearly demonstrated *motivation* for upskilling in this area by dermatologists. Furthermore, results on confidence in core genetic testing tasks are useful in guiding emphasis in training materials to boost clinician *capability*. However, training initiatives are only useful if there is a similar focus on *opportunity—*this may be difficult given the majority of respondents report having insufficient resources to offer patients genetic testing. Future education and training interventions for mainstreaming genetic testing into dermatological practice would benefit from comprehensive consideration of factors affecting opportunity (Damschroder et al., 2009; Proctor et al., 2011).

This report has described genetic testing for familial melanoma as a practical example of testing that could be provided by dermatologists. Provision of guidelines regarding eligibility criteria, consenting process, test ordering and interpretation of test results would be beneficial to increase clinician confidence and uptake.

### 4.1 Strengths and limitations

The generalisability of the survey results is strengthened by the fact that all Australians dermatologists were surveyed, and a high response rate (56%). This is comparably higher than some recent genomic medicine surveys to non-genetic health professionals (11%–26%) (Douma et al., 2016; Carroll et al., 2019). However, the study is still subject to response bias, as those with previous experience/interest in genetics would be more likely to respond. The survey benefited from using previously validated items which allowed comparison with similar studies. However, purpose-designed survey questions on preferences for learning modalities presents decreased generalisability as only 55% of respondents correctly completed the question. The results reported for variables associated with dermatologists’ confidence did not include multiple regression due to small sample size of subgroups and therefore vulnerable to confounders. Furthermore, the study evaluated confidence as opposed to objectively measuring dermatologists’ knowledge and competence. Another limitation to the survey was restricting the population to fully-accredited members of the ACD only. Including trainees would have provided insights into recent changes in education in genetics and any resulting impact on attitudes. Furthermore, including GPs who work full time in skin cancer screening clinics would have been valuable. This is particularly relevant in Australia where, unlike other countries, it is not uncommon for melanomas to be managed entirely within the primary care setting (Smith et al., 2020).

## 5 Conclusion

Australian dermatologists perceive genetic testing as increasingly relevant to practice. However, they currently report low confidence in ordering tests and interpreting results. Having received continuing education on genetics/genomics was found to positively predict genetic confidence. We also found a strong demand for additional resources to enable the upskilling of dermatologists in how to provide genetic testing. This study has generated new evidence to help inform the future implementation of genetic testing into routine dermatological practice in Australia.

## Data Availability

The raw data supporting the conclusions of this article will be made available by the authors, without undue reservation.

## References

[B1] Abdo J. F. Sharma A. Sharma R. (2020). Role of heredity in melanoma susceptibility: A primer for the practicing surgeon. Surg. Clin. North Am. 100 (1), 13–28. 10.1016/j.suc.2019.09.006 31753108

[B2] Aoude L. G. Wadt K. A. Pritchard A. L. Hayward N. K. (2015). Genetics of familial melanoma: 20 years after CDKN2A. Pigment. Cell Melanoma Res. 28 (2), 148–160. 10.1111/pcmr.12333 25431349

[B3] Aspinwall L. G. Leaf S. L. Dola E. R. Kohlmann W. Leachman S. A. (2008). CDKN2A/p16 genetic test reporting improves early detection intentions and practices in high-risk melanoma families. Cancer Epidemiol. Biomarkers Prev. 17 (6), 1510–1519. 10.1158/1055-9965.EPI-08-0010 18559569

[B4] Aspinwall L. G. Taber J. M. Kohlmann W. Leaf S. L. Leachman S. A. (2014). Unaffected family members report improvements in daily routine sun protection 2 years following melanoma genetic testing. Genet. Med. 16 (11), 846–853. 10.1038/gim.2014.37 24763292 PMC4209010

[B5] Badenas C. Aguilera P. Puig-Butillé J. A. Carrera C. Malvehy J. Puig S. (2012). Genetic counseling in melanoma. Dermatol. Ther. 25 (5), 397–402. 10.1111/j.1529-8019.2012.01499.x 23046018 PMC3470473

[B6] Bengtsson M. (2016). How to plan and perform a qualitative study using content analysis. NursingPlus Open 2, 8–14. 10.1016/j.npls.2016.01.001

[B7] Best S. Long J. C. Gaff C. Braithwaite J. Taylor N. (2021). Investigating the adoption of clinical genomics in Australia. An implementation science case study. Genes (Basel) 12 (2), 317. 10.3390/genes12020317 33672413 PMC7926693

[B8] Bouhnik A. D. N'Diaye K. Evans D. G. Harris H. Tibben A. van Asperen C. (2017). Validation of a scale for assessing attitudes towards outcomes of genetic cancer testing among primary care providers and breast specialists. PLoS One 12 (6), e0178447. 10.1371/journal.pone.0178447 28570656 PMC5453525

[B9] Box N. F. Duffy D. L. Chen W. Stark M. Martin N. G. Sturm R. A. (2001). MC1R genotype modifies risk of melanoma in families segregating CDKN2A mutations. Am. J. Hum. Genet. 69 (4), 765–773. 10.1086/323412 11500805 PMC1226062

[B10] Brittain H. K. Scott R. Thomas E. (2017). The rise of the genome and personalised medicine. Clin. Med. 17 (6), 545–551. 10.7861/clinmedicine.17-6-545 PMC6297695 29196356

[B11] Carroll J. C. Allanson J. Morrison S. Miller F. A. Wilson B. J. Permaul J. A. (2019). Informing integration of genomic medicine into primary care: An assessment of current practice, attitudes, and desired resources. Front. Genet. 10 (1189), 1189. 10.3389/fgene.2019.01189 31824576 PMC6882282

[B12] Carroll J. C. Rideout A. L. Wilson B. J. Allanson J. M. Blaine S. M. Esplen M. J. (2009). Genetic education for primary care providers: Improving attitudes, knowledge, and confidence. Can. Fam. Physician 55 (12), e92–e99. 20008584 PMC2793208

[B13] Casebeer L. Brown J. Roepke N. Grimes C. Henson B. Palmore R. (2010). Evidence-based choices of physicians: A comparative analysis of physicians participating in internet CME and non-participants. BMC Med. Educ. 10, 42. 10.1186/1472-6920-10-42 20537144 PMC2892500

[B14] Chow-White P. Ha D. Laskin J. (2017). Knowledge, attitudes, and values among physicians working with clinical genomics: A survey of medical oncologists. Hum. Resour. Health 15 (1), 42. 10.1186/s12960-017-0218-z 28655303 PMC5488429

[B16] Collins F. S. McKusick V. A. (2001). Implications of the human genome project for medical science. Jama 285 (5), 540–544. 10.1001/jama.285.5.540 11176855

[B17] Cornel M. C. (2019). Evidence-based genetic education of non-genetic-expert physicians: Experiences over three decades in amsterdam. Front. Genet. 10 (712), 712. 10.3389/fgene.2019.00712 31428139 PMC6687771

[B18] Corp I. (2020). IBM SPSS statistics for macintosh. 0 edn. NY: Armonk, 27. In.,

[B19] Crellin E. McClaren B. Nisselle A. Best S. Gaff C. Metcalfe S. (2019). Preparing medical specialists to practice genomic medicine: Education an essential part of a broader strategy. Front. Genet. 10, 789. 10.3389/fgene.2019.00789 31572433 PMC6749815

[B20] Culver J. O. Hull J. L. Dunne D. F. Burke W. (2001). Oncologists' opinions on genetic testing for breast and ovarian cancer. Genet. Med. 3 (2), 120–125. 10.1097/00125817-200103000-00006 11280949

[B21] Damschroder L. J. Aron D. C. Keith R. E. Kirsh S. R. Alexander J. A. Lowery J. C. (2009). Fostering implementation of health services research findings into practice: A consolidated framework for advancing implementation science. Implement. Sci. 4, 50. 10.1186/1748-5908-4-50 19664226 PMC2736161

[B22] DeLuca J. Selig D. Poon L. Livezey J. Oliver T. Barrett J. (2020). Toward personalized medicine implementation: Survey of military medicine providers in the area of pharmacogenomics. Mil. Med. 185 (3-4), 336–340. 10.1093/milmed/usz419 31786583

[B23] Demeshko A. Pennisi D. J. Narayan S. Gray S. W. Brown M. A. McInerney-Leo A. M. (2020). Factors influencing cancer genetic somatic mutation test ordering by cancer physician. J. Transl. Med. 18 (1), 431. 10.1186/s12967-020-02610-7 33183308 PMC7663861

[B24] Diamonstein C. Stevens B. Shahrukh Hashmi S. Refuerzo J. Sullivan C. Hoskovec J. (2018). Physicians’ awareness and utilization of genetic services in Texas. J. Genet. Couns. 27 (4), 968–977. 10.1007/s10897-017-0199-z 29280038

[B25] Douma K. F. L. Smets E. M. A. Allain D. C. (2016). Non-genetic health professionals’ attitude towards, knowledge of and skills in discussing and ordering genetic testing for hereditary cancer. Fam. Cancer 15 (2), 341–350. 10.1007/s10689-015-9852-6 26590592 PMC4803807

[B26] Duffy D. L. Lee K. J. Jagirdar K. Pflugfelder A. Stark M. S. McMeniman E. K. (2019). High naevus count and MC1R red hair alleles contribute synergistically to increased melanoma risk. Br. J. Dermatol. 181 (5), 1009–1016. 10.1111/bjd.17833 30820946

[B27] Freeley M. (2020). Current postgraduate training programs and online courses in precision medicine. Expert Rev. Mol. diagn. 20 (6), 569–574. 10.1080/14737159.2020.1709826 31875486

[B28] Frost C. J. Andrulis I. L. Buys S. S. Hopper J. L. John E. M. Terry M. B. (2019). Assessing patient readiness for personalized genomic medicine. J. Community Genet. 10 (1), 109–120. 10.1007/s12687-018-0365-5 29804257 PMC6325047

[B29] George A. Riddell D. Seal S. Talukdar S. Mahamdallie S. Ruark E. (2016). Implementing rapid, robust, cost-effective, patient-centred, routine genetic testing in ovarian cancer patients. Sci. Rep. 6, 29506. 10.1038/srep29506 27406733 PMC4942815

[B30] Gordon L. G. Rowell D. (2015). Health system costs of skin cancer and cost-effectiveness of skin cancer prevention and screening: A systematic review. Eur. J. Cancer Prev. 24 (2), 141–149. 10.1097/CEJ.0000000000000056 25089375

[B31] Guitera P. Menzies S. W. Coates E. Azzi A. Fernandez-Penas P. Lilleyman A. (2021). Efficiency of detecting new primary melanoma among individuals treated in a high-risk clinic for skin surveillance. JAMA Dermatol. 157 (5), 521–530. 10.1001/jamadermatol.2020.5651 33729464 PMC7970391

[B32] Haga S. B. Kim E. Myers R. A. Ginsburg G. S. (2019). Primary care physicians' knowledge, attitudes, and experience with personal genetic testing. J. Pers. Med. 9 (2), E29. 10.3390/jpm9020029 31137623 PMC6617198

[B33] Harding B. Webber C. Rühland L. Dalgarno N. Armour C. Birtwhistle R. (2019). Bridging the gap in genetics: A progressive model for primary to specialist care. BMC Med. Educ. 19 (1), 195. 10.1186/s12909-019-1622-y 31185964 PMC6558677

[B34] Heald B. Plesec T. Liu X. Pai R. Patil D. Moline J. (2013). Implementation of universal microsatellite instability and immunohistochemistry screening for diagnosing lynch syndrome in a large academic medical center. J. Clin. Oncol. 31 (10), 1336–1340. 10.1200/JCO.2012.45.1674 23401454 PMC4878100

[B35] Hoskovec J. M. Bennett R. L. Carey M. E. DaVanzo J. E. Dougherty M. Hahn S. E. (2018). Projecting the supply and demand for certified genetic counselors: A workforce study. J. Genet. Couns. 27 (1), 16–20. 10.1007/s10897-017-0158-8 29052810

[B36] James K. M. Ziegenfuss J. Y. Tilburt J. C. Harris A. M. Beebe T. J. (2011). Getting physicians to respond: The impact of incentive type and timing on physician survey response rates. Health Serv. Res. 46, 232–242. 10.1111/j.1475-6773.2010.01181.x 20880042 PMC3034272

[B37] Johnson K. B. Clayton E. W. Starren J. Peterson J. (2020). The implementation chasm hindering genome-informed health care. J. Law Med. Ethics 48 (1), 119–125. 10.1177/1073110520916999 PMC7395963 32342791

[B38] Johnson L-M. Valdez J. M. Quinn E. A. Sykes A. D. McGee R. B. Nuccio R. (2017). Integrating next-generation sequencing into pediatric oncology practice: An assessment of physician confidence and understanding of clinical genomics. Cancer 123 (12), 2352–2359. 10.1002/cncr.30581 28192596 PMC5710798

[B39] Kathrens-Gallardo A. Propst L. Linn E. Pothast R. Wicklund C. Arjunan A. (2021). OB/GYN residents' training, attitudes, and comfort level regarding genetics. J. Assist. Reprod. Genet. 38, 2871–2880. 10.1007/s10815-021-02310-1 34515898 PMC8608976

[B40] Kemp Z. Turnbull A. Yost S. Seal S. Mahamdallie S. Poyastro-Pearson E. (2019). Evaluation of cancer-based criteria for use in mainstream BRCA1 and BRCA2 genetic testing in patients with breast cancer. JAMA Netw. Open 2 (5), e194428. 10.1001/jamanetworkopen.2019.4428 31125106 PMC6632150

[B41] Kentwell M. Dow E. Antill Y. Wrede C. D. McNally O. Higgs E. (2017). Mainstreaming cancer genetics: A model integrating germline BRCA testing into routine ovarian cancer clinics. Gynecol. Oncol. 145 (1), 130–136. 10.1016/j.ygyno.2017.01.030 28162234

[B42] Kohut K. Limb S. Crawford G. (2019). The changing role of the genetic counsellor in the genomics era. Curr. Genet. Med. Rep. 7 (2), 75–84. 10.1007/s40142-019-00163-w

[B43] Leachman S. A. Lucero O. M. Sampson J. E. Cassidy P. Bruno W. Queirolo P. (2017). Identification, genetic testing, and management of hereditary melanoma. Cancer Metastasis Rev. 36 (1), 77–90. 10.1007/s10555-017-9661-5 28283772 PMC5385190

[B44] Maas E. J. Betz-Stablein B. Aoude L. G. Soyer H. P. McInerney-Leo A. M. (2022). Unusual suspects in hereditary melanoma: POT1, POLE, BAP1. Trends Genet. S0168-9525 (22), online ahead of print. 10.1016/j.tig.2022.06.007 35811174

[B45] McClaren B. J. King E. A. Crellin E. Gaff C. Metcalfe S. A. Nisselle A. (2020). Development of an evidence-based, theory-informed national survey of physician preparedness for genomic medicine and preferences for genomics continuing education. Front. Genet. 11 (59), 59. 10.3389/fgene.2020.00059 32194615 PMC7063665

[B46] McCuaig J. M. Armel S. R. Care M. Volenik A. Kim R. H. Metcalfe K. A. (2018). Next-Generation service delivery: A scoping review of patient outcomes associated with alternative models of genetic counseling and genetic testing for hereditary cancer. Cancers (Basel) 10 (11), E435. 10.3390/cancers10110435 30428547 PMC6266465

[B47] Michie S. van Stralen M. M. West R. (2011). The behaviour change wheel: A new method for characterising and designing behaviour change interventions. Implement. Sci. 6 (1), 42. 10.1186/1748-5908-6-42 21513547 PMC3096582

[B48] Miesfeldt S. Feero W. G. Lucas F. L. Rasmussen K. (2018). Association of patient navigation with care coordination in an Lynch syndrome screening program. Transl. Behav. Med. 8 (3), 450–455. 10.1093/tbm/ibx078 29800403

[B49] Murphy M. J. (2015). Attitudes concerning clinical molecular testing among dermatology trainees at a single institution. Am. J. Dermatopathol. 37 (7), 590. 10.1097/DAD.0000000000000136 25738467

[B50] Nippert I. Harris H. J. Julian-Reynier C. Kristoffersson U. Ten Kate L. P. Anionwu E. (2011). Confidence of primary care physicians in their ability to carry out basic medical genetic tasks-a European survey in five countries-Part 1. J. Community Genet. 2 (1), 1–11. 10.1007/s12687-010-0030-0 22109718 PMC3186019

[B51] Nippert I. Harris H. J. Julian-Reynier C. Kristoffersson U. ten Kate L. P. Anionwu E. (2011). Confidence of primary care physicians in their ability to carry out basic medical genetic tasks—A European survey in five countries—Part 1. J. Community Genet. 2 (1), 1–11. 10.1007/s12687-010-0030-0 22109718 PMC3186019

[B52] Nisselle A. King E. A. McClaren B. Janinski M. Metcalfe S. Gaff C. (2021). Measuring physician practice, preparedness and preferences for genomic medicine: A national survey. BMJ Open 11 (7), e044408. 10.1136/bmjopen-2020-044408 PMC8273463 34244249

[B53] Noel H. Huang A. R. (2019). The effect of varying incentive amounts on physician survey response. Eval. Health Prof. 42 (1), 71–81. 10.1177/0163278718809844 30384767

[B54] O'Shea R. Taylor N. Crook A. Jacobs C. Jung Kang Y. Lewis S. (2021). Health system interventions to integrate genetic testing in routine oncology services: A systematic review. PLoS One 16 (5), e0250379. 10.1371/journal.pone.0250379 34010335 PMC8133413

[B55] Plaskocinska I. Shipman H. Drummond J. Thompson E. Buchanan V. Newcombe B. (2016). New paradigms for BRCA1/BRCA2 testing in women with ovarian cancer: Results of the genetic testing in epithelial ovarian cancer (GTEOC) study. J. Med. Genet. 53 (10), 655–661. 10.1136/jmedgenet-2016-103902 27208206 PMC5099175

[B56] Potrony M. Badenas C. Aguilera P. Puig-Butille J. A. Carrera C. Malvehy J. (2015). Update in genetic susceptibility in melanoma. Ann. Transl. Med. 3 (15), 210. 10.3978/j.issn.2305-5839.2015.08.11 26488006 PMC4583600

[B15] Primiero C. A. Finnane A. Yanes T. Peach B. Soyer H. P. (2022). Protocol to evaluate a pilot program to upskill clinicians in providing genetic testing for familial melanoma. PLOS One. in press/forthcoming. 10.1371/journal.pone.0275926 PMC9728910 36477719

[B57] Primiero C. A. Yanes T. Finnane A. Soyer H. P. McInerney-Leo A. M. (2021). A systematic review on the impact of genetic testing for familial melanoma I: Primary and secondary preventative behaviours. Dermatology 237 (5), 806–815. 10.1159/000513919 33588421

[B58] Primiero C. A. Yanes T. Finnane A. Soyer H. P. McInerney-Leo A. M. (2021). A systematic review on the impact of genetic testing for familial melanoma II: Psychosocial outcomes and attitudes. Dermatology 237 (5), 816–826. 10.1159/000513576 33508831

[B59] Proctor E. Silmere H. Raghavan R. Hovmand P. Aarons G. Bunger A. (2011). Outcomes for implementation research: Conceptual distinctions, measurement challenges, and research agenda. Adm. Policy Ment. Health 38 (2), 65–76. 10.1007/s10488-010-0319-7 20957426 PMC3068522

[B60] Rahman B. Lanceley A. Kristeleit R. S. Ledermann J. A. Lockley M. McCormack M. (2019). Mainstreamed genetic testing for women with ovarian cancer: First-year experience. J. Med. Genet. 56 (3), 195–198. 10.1136/jmedgenet-2017-105140 29535157

[B61] Read J. Wadt K. A. Hayward N. K. (2016). Melanoma genetics. J. Med. Genet. 53 (1), 1–14. 10.1136/jmedgenet-2015-103150 26337759

[B62] Reed E. K. Johansen Taber K. A. Ingram Nissen T. Schott S. Dowling L. O. O'Leary J. C. (2016). What works in genomics education: Outcomes of an evidenced-based instructional model for community-based physicians. Genet. Med. 18 (7), 737–745. 10.1038/gim.2015.144 26583682

[B63] Rees J. (1992). Forward dermatology. Bmj 304 (6827), 590. 10.1136/bmj.304.6827.590 1313720 PMC1881326

[B64] Ribeiro Moura Brasil Arnaut J. Dos Santos Guimarães I. Evangelista Dos Santos A. C. de Moraes Lino da Silva F. Machado J. R. de Melo A. C. (2021). Molecular landscape of hereditary melanoma. Crit. Rev. Oncol. Hematol. 164, 103425. 10.1016/j.critrevonc.2021.103425 34245855

[B65] Richardson M. Min H. J. Hong Q. Compton K. Mung S. W. Lohn Z. (2020). Oncology clinic-based hereditary cancer genetic testing in a population-based health care system. Cancers (Basel) 12 (2), E338. 10.3390/cancers12020338 32028617 PMC7072228

[B66] Rothstein M. A. (2018). Time to end the use of genetic test results in life insurance underwriting. J. Law Med. Ethics 46 (3), 794–801. 10.1177/1073110518804243 30336088 PMC8607993

[B67] Rumford M. Lythgoe M. McNeish I. Gabra H. Tookman L. Rahman N. (2020). Oncologist-led BRCA 'mainstreaming' in the ovarian cancer clinic: A study of 255 patients and its impact on their management. Sci. Rep. 10 (1), 3390. 10.1038/s41598-020-60149-5 32098980 PMC7042365

[B68] Scheuner M. T. Wang S. J. Raffel L. J. Larabell S. K. Rotter J. I. (1997). Family history: A comprehensive genetic risk assessment method for the chronic conditions of adulthood. Am. J. Med. Genet. 71 (3), 315–324. 10.1002/(sici)1096-8628(19970822)71:3<315:aid-ajmg12>3.0.co;2-n 9268102

[B69] Shagalov D. R. Ferzli G. M. Wildman T. Glick S. A. (2017). Genetic testing in dermatology: A survey analyzing obstacles to appropriate care. Pediatr. Dermatol. 34 (1), 33–38. 10.1111/pde.12981 27653748

[B70] Smith A. L. Watts C. G. Robinson S. Schmid H. Chang C. H. Thompson J. F. (2020). GPs' involvement in diagnosing, treating, and referring patients with suspected or confirmed primary cutaneous melanoma: A qualitative study. BJGP Open 4 (2), bjgpopen20X101028. 10.3399/bjgpopen20X101028 PMC7330208 32295791

[B71] Stump T. K. Aspinwall L. G. Drummond D. M. Taber J. M. Kohlmann W. Champine M. (2020). CDKN2A testing and genetic counseling promote reductions in objectively measured sun exposure one year later. Genet. Med. 22 (1), 26–34. 10.1038/s41436-019-0608-9 31371819 PMC6946876

[B72] Talwar D. Tseng T-S. Foster M. Xu L. Chen L-S. (2017). Genetics/genomics education for nongenetic health professionals: A systematic literature review. Genet. Med. 19 (7), 725–732. 10.1038/gim.2016.156 27763635

[B73] Tiller J. McInerney-Leo A. Belcher A. Boughtwood T. Gleeson P. Delatycki M. (2021). Study protocol: The Australian genetics and life insurance moratorium-monitoring the effectiveness and response (A-GLIMMER) project. BMC Med. Ethics 22 (1), 63. 10.1186/s12910-021-00634-2 34020638 PMC8138092

[B74] Tiller J. Morris S. Rice T. Barter K. Riaz M. Keogh L. (2020). Genetic discrimination by Australian insurance companies: A survey of consumer experiences. Eur. J. Hum. Genet. 28 (1), 108–113. 10.1038/s41431-019-0426-1 31281182 PMC6906286

[B75] Tiller J. Otlowski M. Lacaze P. (2017). Should Australia ban the use of genetic test results in life insurance? Front. Public Health 5 (330), 330. 10.3389/fpubh.2017.00330 29322039 PMC5733354

[B76] Tiller J. M. Keogh L. A. McInerney-Leo A. M. Belcher A. Barlow-Stewart K. Boughtwood T. (2021). A step forward, but still inadequate: Australian health professionals' views on the genetics and life insurance moratorium. J. Med. Genet. 59, 817–826. 10.1136/jmedgenet-2021-107989 34544841

[B77] Torre K. Russomanno K. Ferringer T. Elston D. Murphy M. J. (2018). Educational gaps in molecular diagnostics, genomics, and personalized medicine in dermatopathology training: A survey of U.S. Dermatopathology fellowship program directors. Am. J. Dermatopathol. 40 (1), 43–48. 10.1097/DAD.0000000000000909 28509701

[B78] Toussi A. Mans N. Welborn J. Kiuru M. (2020). Germline mutations predisposing to melanoma. J. Cutan. Pathol. 47 (7), 606–616. 10.1111/cup.13689 32249949 PMC8232041

[B79] Williams M. S. (2019). Early lessons from the implementation of genomic medicine programs. Annu. Rev. Genomics Hum. Genet. 20, 389–411. 10.1146/annurev-genom-083118-014924 30811224

[B80] Zhou A. E. Hoegler K. M. Solimine J. F. (2021). Genetic counseling and testing for hereditary causes of melanoma can lead to earlier detection of skin cancer and other malignancies. Int. J. Dermatol. 61, e233–e234. 10.1111/ijd.15716 34138469

