# Supplementary material for: Attitudes of Australian dermatologists on the use of genetic testing: A cross-sectional survey with a focus on melanoma
Source: Front Genet. 2022 Oct 24;13:919134. doi: 10.3389/fgene.2022.919134 (PMC9638172; doi:10.3389/fgene.2022.919134)
Supplement: Supplementary file 1 [file DataSheet1.DOCX]

**Section 1: ATTITUDES TOWARD UTILITY OF GENETIC TESTING**

1. **Please rate your agreement with the following statements.**

|  | Strongly Agree |  |  |  | Strongly Disagree |
| --- | --- | --- | --- | --- | --- |
| Genetic Testing is relevant to my practice today | **1** | **2** | **3** | **4** | **5** |
| Genetic Testing will become increasingly relevant in the future | **1** | **2** | **3** | **4** | **5** |

**Section 2: COMFORT AND CONFIDENCE**

1. **Please indicate which of the following statements align with your perception of genetic testing in dermatology**

|  | Agree | Disagree |
| --- | --- | --- |
| Genetic testing for melanoma could be of value to the patient |  |  |
| Genetic testing for melanoma could be of value to the patient’s family |  |  |
| Genetic testing for melanoma could be of value in informing management decisions |  |  |
| Genetic testing for melanoma could be of value in improving primary/secondary prevention |  |  |
| Genetic testing for melanoma could limit the patient’s private health insurance coverage |  |  |
| Genetic testing for melanoma could limit the patient’s life insurance coverage |  |  |
| Are you aware that there was a change in policy in July 2019, and a moratorium was introduced on the use of genetic testing in life insurance underwriting? |  |  |
| Genetic testing for melanoma could stigmatise the patients as a “worried well” person |  |  |

1. **Please indicate which of the following tasks you have or have not performed as a dermatologist, for any dermatological condition.**

|  | Yes | No |
| --- | --- | --- |
| I have discussed genetic testing with my patients |  |  |
| I have offered genetic testing to my patients |  |  |
| I have ordered genetic testing for at least one of my patients |  |  |
| I referred my patients to clinical genetics services to pursue genetic testing |  |  |
| I have the necessary services and staff to offer my patients genetic testing |  |  |

1. **Please indicate your level of confidence in the following skills by selecting the number that corresponds.**

|  | Not at all confident |  |  | |  | | Very confident |
| --- | --- | --- | --- | --- | --- | --- | --- |
| Collecting a family history | **1** | **2** | | **3** | | **4** | **5** |
| Ability to identify patients at increased risk for a dermatological condition based on family history | **1** | **2** | | **3** | | **4** | **5** |
| Ability to obtain informed consent (for genetic testing) | **1** | **2** | | **3** | | **4** | **5** |
| Ability to identify the appropriate genetic test for my patient | **1** | **2** | | **3** | | **4** | **5** |
| Ability to order genetic testing | **1** | **2** | | **3** | | **4** | **5** |
| Ability to interpret genetic test results | **1** | **2** | | **3** | | **4** | **5** |
| Ability to use genetic information in management decisions | **1** | **2** | | **3** | | **4** | **5** |
| Ability to identify patients that could benefit from genetic counselling | **1** | **2** | | **3** | | **4** | **5** |
| Ability to educate my patients about inheritance and risk of genetic diseases | **1** | **2** | | **3** | | **4** | **5** |
| Ability to educate my patients about the genetic aetiology/mechanism of disease | **1** | **2** | | **3** | | **4** | **5** |
| Ability to discuss possible outcomes of genetic testing | **1** | **2** | | **3** | | **4** | **5** |

**Section 3: EDUCATION**

1. **Below is a list of activities that can be used to upskill in genomic medicine. Please rank which activities would be MOST helpful to keep up to date with, or learn new skills in, genomic medicine. (1 = most helpful | 5 = least helpful)**

|  | Rank activities 1 to 5 |
| --- | --- |
| Experiential (observation/immersion in cases) |  |
| Face-to-face courses/tutorials |  |
| Online courses/tutorials |  |
| Printed materials |  |
| A “hotline” to talk to genetics professional |  |

1. **Please indicate if you have completed continued education in genetics.**

*If yes is selected, how useful was the activity in your understanding of genetic testing in practice*

|  | Yes | No | |
| --- | --- | --- | --- |
| Have you ever completed a unit of study on genetics/genomics as part of an award course (degree/certificate/diploma)? |  |  | |
| If Yes, how useful was it?  1  10  Not at all useful Very useful  Yes No . | | | |
| Have you ever completed a short course on genetics/genomics?  1  10 |  |  | |
| If Yes, how useful was it?  Not at all useful Very useful  Yes No . | | | |
| Have you ever had training regarding the ethical considerations of genetic/genomic testing or research? |  | |  |
| If Yes, how useful was it?  1  10  Not at all useful Very useful | | | |

**Section 4: DEMOGRAPHICS**

| 1. **Where are you located?** *Select one option* | | | | | | | | | |
| --- | --- | --- | --- | --- | --- | --- | --- | --- | --- |
| Australia | | | | | | | | | |
| - ACT | - NSW | - NT | | - QLD | - SA | - TAS | | - VIC | - WA |
| - New Zealand | | | | | - Other | | | | |
| 1. **How many years has it been since you graduated from medical school?** | | | | | | | | | |
| - 1 – 10 years | | | - 11 – 20 years | | | | - 21 – 30 years | | |
| - 31 – 40 years | | | - >40 years | | | |  | | |
| 1. **Do you have Sub-Speciality (e.g. Paediatric Dermatology)?** | | | | | | | | | |
| - Yes | | | - No | | | |  | | |
| If Yes, what type?  _____________________________ | | |  | | | |  | | |
| 1. **How often do you order a genetic/genomic test?** | | | | | | | | | |
| - Never | | | - Rarely (≤5 times a year) | | | | - Often (≥ once a month) | | |
| - Routinely (≥ once a week) | | |  | | | |  | | |
|  | | |  | | | |  | | |

- 1. If Never or Rarely, why not? (tick all that apply)
- Not relevant to my practice
- I do not feel confident
- It is not my role
- I do not have access to a genetic service
- I do not have time
- If other, please specify: __________________________

1. **Have patients ever initiated a conversation about genetic testing?**

| - Yes | - No |
| --- | --- |

Do you have any additional thoughts regarding genetic testing in dermatology, which you would like to share?

**Thank you!**

Results of the survey will be shared with and disseminated by the Australian College of Dermatologists.

***Thank you for taking the time to complete this survey. Your responses are invaluable in shaping the future training of the profession.***
